# A recent duplication revisited: phylogenetic analysis reveals an ancestral duplication highly-conserved throughout the *Oryza *genus and beyond

**DOI:** 10.1186/1471-2229-9-146

**Published:** 2009-12-10

**Authors:** Julie Jacquemin, Michèle Laudié, Richard Cooke

**Affiliations:** 1 Laboratoire Génome et Développement des Plantes, Unité mixte de recherche 5096, Centre national de la recherche scientifique, Institut pour la recherche et le développement, Université de Perpignan via Domitia, 58, Av Paul Alduy, 66860 Perpignan Cedex, France

## Abstract

**Background:**

The role of gene duplication in the structural and functional evolution of genomes has been well documented. Analysis of complete rice (*Oryza sativa*) genome sequences suggested an ancient whole genome duplication, common to all the grasses, some 50-70 million years ago and a more conserved segmental duplication between the distal regions of the short arms of chromosomes 11 and 12, whose evolutionary history is controversial.

**Results:**

We have carried out a comparative analysis of this duplication within the wild species of the genus *Oryza*, using a phylogenetic approach to specify its origin and evolutionary dynamics. Paralogous pairs were isolated for nine genes selected throughout the region in all *Oryza *genome types, as well as in two outgroup species, *Leersia perrieri *and *Potamophila parviflora*. All *Oryza *species display the same global evolutionary dynamics but some lineage-specific features appear towards the proximal end of the duplicated region. The same level of conservation is observed between the redundant copies of the tetraploid species *Oryza minuta*. The presence of orthologous duplicated blocks in the genome of the more distantly-related species, *Brachypodium distachyon*, strongly suggests that this duplication between chromosomes 11 and 12 was formed as part of the whole genome duplication common to all Poaceae.

**Conclusion:**

Our observations suggest that recurrent but heterogeneous concerted evolution throughout the *Oryza *genus and in related species has led specifically to the extremely high sequence conservation occurring in this region of more than 2 Mbp.

## Background

The analysis of an increasing number of complete genome sequences has allowed in-depth studies of the role of sequence redundancy in genome evolution [1-4]. Gene duplication has been considered for a long time to be a source of novel functions, and to have played a significant part in genome functional evolution and species divergence. Hypotheses on the evolution of genes duplicated by whole genome duplication (WGD), segmental or local events were proposed in 1970 by Ohno [5] and models for the evolution of these duplicated genes have since been elaborated. Following the unexpected observation that *Arabidopsis thaliana *is a paleopolyploid, a whole genome duplication (WGD) having occurred some 35-40 million years ago (MYA) [6], it was shown that extant plant genomes probably all result from successive cycles of WGD/diploidization [7]. Major losses [8,9], structural and functional divergence [10,11] or concerted evolution [12], have all been observed in eukaryotic genomes, in particular after whole genome duplication events.

Analysis of the complete sequences of the genomes of rice (*Oryza sativa*) subspecies *indica *and *japonica *suggested two independent duplications: a WGD that occurred between 53 and 94MYA, and which is thus common to all cereals, and a more recent segmental duplication between the distal regions of the short arms of chromosomes 11 and 12 [13]. The relative chronology of this latter duplication and speciation events within the *Oryza *genus are unclear. It was first identified by genetic [14] and physical mapping [15], with an estimated length of 2.5-3 Mbp. Other authors, using synonymous substitution rates between conserved gene pairs for dating, described a duplication of 5.44 (chromosome 11) and 4.27 Mbp (chromosome 12) 5MYA [8], 3.3 Mbp 7.7MYA [16], 3.3 Mbp 20MYA [17] or 6.5 and 4.8 Mbp 21MYA [13], while Goff *et al*. [18] calculated 25MYA using protein/protein alignments. According to Gaut [19], the divergence between Erhartoideae (*Oryza*) and the other Pooideae (such as wheat) is approximately 46MYA. Molecular dating places the divergence of the *Oryza-Leersia *clade with other genera at ~20MYA, that of the *Oryza *and *Leersia *genera at 14.2MYA, and divergence of the most basal species in the genus (*O. granulata*) at ~10MYA [20], in agreement with fossil reports [21]. Recent data using other genes has confirmed this divergence time [22]. The evolutionary dynamics of the duplication have been studied between the two subspecies *O. sativa *ssp. *japonica *and *O. sativa *ssp. *indica *[23]. These authors concluded that this region could be affected by concerted evolution.

Previous studies on the evolution of large-scale gene duplication were based on the available genome sequences from widely-divergent species and little is known about the short-term evolution of duplicated copies and their role in species divergence within a genus. The model species *Oryza sativa *L. and its wild relatives represent an ideal system to answer questions about gene and genome evolution [24,25]. Genomic data and the well-characterized phylogeny available for this genus enable a comparative approach of the evolutionary history of this duplication between several closely-related species.

Adopting a phylogenetic approach, we isolated and sequenced orthologous duplicated pairs from the region of interest in a set of 7 representative *Oryza *genomes, including tetraploid *O. minuta *and the surrogate parental species *O. punctata *and *O. officinalis*, as well as in the closely-related species *Leersia perrieri *and *Potamophila parviflora*. We demonstrate the presence and strong conservation of the duplication both within the genus and in close outgroup species. Its presence in the more distant species *Brachypodium distachyon *and *Sorghum bicolor *[26] suggests that its origin is concomitant with the cereal ancestral genome duplication and that the specific mechanisms that have led to the high levels of sequence conservation within this region of the *Oryza *genomes are probably recurrent.

## Results

### Sequence conservation in subtelomeric regions of chromosomes 11 and 12

The duplicated subtelomeric regions of *Oryza sativa *ssp. *japonica *chromosomes 11 and 12 have been described as being highly conserved [8,13,17]. Additional file 1 shows a dot plot between the first 2.5 Mbp of these chromosomes. Sequence conservation is particularly high within the first 2 Mbp. Beyond this point, large-scale conservation is no longer detectable, similarity being limited to individual genes or blocks of genes, which are visible on the zoom of this region. The loss of colinearity is due to sequence divergence and the movement of transposable elements since the duplication event.

### Phylogenetic analysis

**Figure 1:**
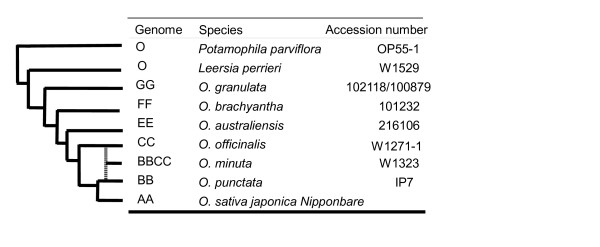
**Phylogenetic relationships, genome type and accessions number of representative *Oryza *species analysed**.

Phylogenetic trees based on duplicated sequences can have two topologies, depending on the relative dates of the duplication and speciation events. If duplication predates speciation, we expect to find one copy of each gene pair from all species in one branch of the tree and the other copies in a second branch. In contrast, if the duplication follows speciation we expect to find the paralogous gene pairs as terminal nodes. If the duplication between chromosomes 11 and 12 occurred within the *Oryza *genus, we would expect to find two gene copies for post-duplication species, with a "speciation after duplication" topology, and only one for species having diverged before the duplication. Using primer pairs selected as described in Methods, we amplified and sequenced gene fragments from seven *Oryza *species and the closely-related *Leersia perrieri *and *Potamophila parviflora *(Figure 1), corresponding to nine genes (named A to I for simplification) selected along the duplicated region (Table 1). Among these, five (B, D, E, H, I) were retained for genus-wide analysis according to the following criteria: minimum length of 500 bp, amplification of both exonic and intronic sequences to clone the more variable intronic regions and their distribution on the duplicated fragment. The four remaining sequences (A, C, F, G) were amplified on a reduced set of species (*O. brachyantha *and/or *O. granulata*, *L. perrieri *and *P. parviflora*). Putative functions were verified by BLASTX alignment against Viridiplantae proteins.

**Table 1:** The nine chromosome 11 and 12 paralogous pairs sequenced in *Oryza *species

*A*	Os11g01154	217	96233-98565	Os12g01160	217	92201-94423	137	Trans-2-enoyl-CoA reductase
***B***	**Os11g01380**	**597**	**233285-243004**	**Os12g01390**	**594**	**248725-258395**	**228**	**Clathrin heavy chain binding**
*C*	Os11g01420	304	253787-251730	Os12g01430	302	269040-266954	92	mRNA turnover protein 4
***D***	**Os11g03050**	**736**	**1053217-1058699**	**Os12g02820**	**735**	**1009926-1005023**	**326**	**Ethanolamine-phosphate cytidylyltransferase**
***E***	**Os11g03730**	**663**	**1453355-1458633**	**Os12g03470**	**680**	**1359377-1365215**	**120**	**Alpha-L-arabinofuranosidase C-terminus**
*F*	Os11g04030	626	1630227-1625740	Os12g03860	427	1587934 -1583402	188	Major facilitator superfamily antiporter
*G*	Os11g04200	494	1711766-1707219	Os12g04010	495	1667882-1663261	297	M-phase phosphoprotein 10
***H***	**Os11g04740**	**1289**	**2020022-2015978**	**Os12g04520**	**1294**	**1925626-1921577**	**283**	**L-Galactono,4-lactone dehydrogenase**
***I***	**Os11g04980**	**880**	**2136990-2128145**	**Os12g04990**	**792**	**2089033-2088254**	**102**	**AMP-binding enzyme family**

The genes in bold were amplified on the complete sample set whereas the others were amplified on the more distant species only. CDS size is given for the multiple alignement of codons sequences.

**Figure 2:**
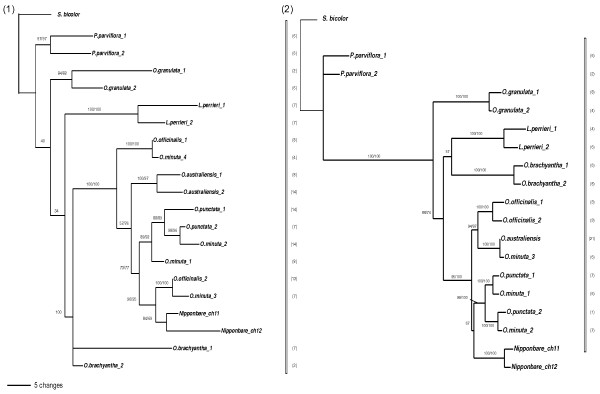
**ML trees inferred from genes B (1) and D (2)**. Numbers above branches indicated bootstrap support for ML and MP respectively. If only one number is present, that means incongruence between the two methods and only the ML bootstrap is shown. Numbers of clones sequenced for each copy are in parentheses. *Oryza minuta *(allotetraploid) copies are underligned.

**Figure 3:**
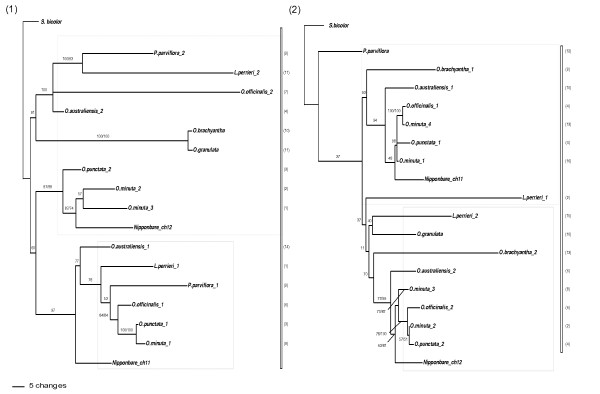
**ML trees inferred from genes E (1) and I (2)**. Numbers above branches indicated bootstrap support for ML and MP respectively. If only one number is present, that means incongruence between the two methods and only the ML bootstrap is shown. Numbers of clones sequenced for each copy are in parentheses. *Oryza minuta *(allotetraploid) copies are underligned.

**Figure 4:**
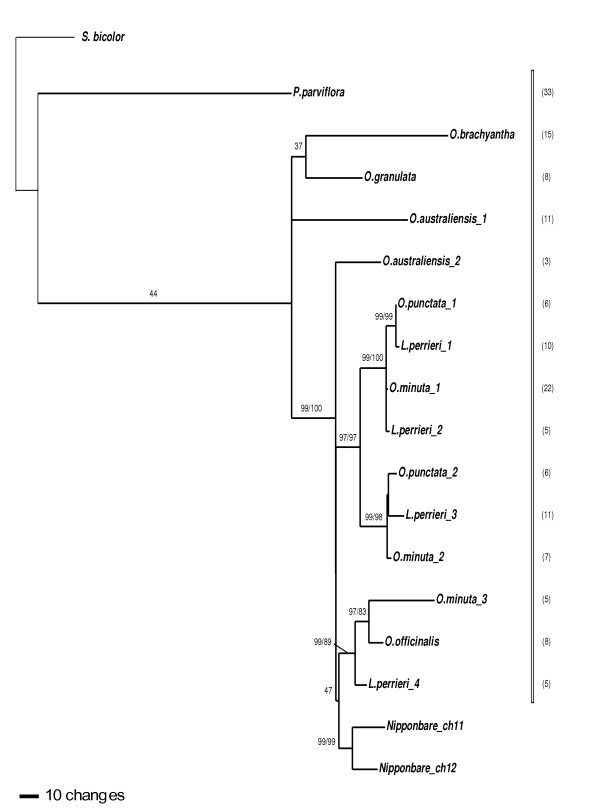
**ML tree inferred from gene H**. Numbers above branches indicated bootstrap support of ML and MP respectively. If only one number is present, that means incongruence between the two methods and only the ML bootstrap is shown. Numbers of clones sequenced for each copy are in parentheses. *Oryza minuta *(allotetraploid) copies are underligned.

Figures 2, 3 and 4 and Additional files 2 and 3 show phylogenetic trees constructed using the maximum likelihood (ML) method, and bootstrap values for both ML and maximum parsimony (MP) analysis. Trees were rooted with homologous copies identified in either *Sorghum bicolor *[26] or *Brachypodium distachyon *http://www.brachypodium.org genomes, but the branches leading to these outgroup are not proportional to their divergence. Two copies of each sequence were isolated almost systematically in all species including *L. perrieri *and *P. parviflora*. The fact that two separate copies were isolated for all genes in *Leersia *is not surprising considering its position in our trees, where all copies of all amplified sequences from this species are grouped in the same clade as the *Oryza *species.

For sequences A, C, F and G all paralogous copies group together, showing a "duplication after speciation" topology, except for the two gene C copies of *O. granulata *(See Additional files 2 and 3: ML trees inferred from genes A (1), C (2), F (3) and G (4)). For sequences F and G, only one copy from *P. parviflora *and *O. granulata*, respectively, were isolated. Bootstrap values are not strong for internal nodes, certainly because of the weak phylogenetic signal for these sequences (Table 2), but terminal nodes grouping the paralogous copies are strongly supported.

**Table 2:** Characteristics of the gene data set for phylogenetic analysis and corresponding GenBank accession numbers

	A	B	C	D	E	F	G	H	I
Mean dS	0.130	**0.040**	0.100	**0.120**	**0.130**	0.150	0.050	**0.080**	**0.090**
Mean dN	0.020	**0.050**	0.010	**0.020**	**0.100**	0.010	0.060	**0.010**	**0.020**
Mean K	0.221	**0.084**	0.234	**0.188**	**0.141**	0.236	0.148	**0.187**	**0.208**
Parsimony informative sites	27	**76**	23	**84**	**150**	68	67	**169**	**104**
DIPs-number of InDel events	6	**43**	35	**83**	**66**	28	17	**106**	**93**
Transition/Transversion ratio	3.72	**2.2**	1.51	**3.2**	**1.1**	3.29	1.17	**3.1**	**0.78**
Accessions [Genbank:FJ958xxx]	202-207	**208-225**	226-233	**234-249**	**250-264**	265-271	272-278	**279-293**	**294-309**

The genes in bold were amplified on the complete sample set whereas the others were amplified on the more distant species only. Mean dS, mean dN and mean K are the average synonymous, non-synonymous and non-coding substitutions rates values for all the pairwise comparisons in one data set.

For sequences B and D (Figure 2), two copies were isolated for all species, and all paralogous pairs are grouped together, except for *O. officinalis *gene B copy 1 which diverged earlier. Their strong conservation rate is reflected by the weak support for internal nodes, particularly for sequence B. For sequence D, we isolated only one copy from *O. australiensis *but, given the number of clones sampled (21), the second copy has probably been deleted or is too divergent to be amplified. Moreover, this single copy is sister to one of the tetraploid *O. minuta *copies, which was not expected. Overall, sequences B and D clearly show a "duplication after speciation" topology type.

On the other hand, sequences E and I display a "duplication before speciation" topology (Figure 3). Only one copy of gene E was isolated for *O. brachyantha *and *O. granulata*, but for all other species the two copies are separated. One copy forms a monophyletic clade with the Nipponbare chromosome 11 sequence, while the second and third copies of *O. punctata *and *O. minuta *are grouped with the Nipponbare paralog on chromosome 12. The second copies from *O. officinalis*, *O. australiensis*, *L. perrieri *and *P. parviflora*, as well as the single copies from *O. brachyantha *and *O. granulata*, are grouped in a second, large clade, being more divergent from the copy 1 in these more ancient species. This is in agreement with the hypothesis of an independent divergence of the two paralogous sequences after duplication, the *O. brachyantha *and *O. granulata *single copies belonging to the "chromosome 12" clade.

In the ML tree of gene I we clearly observe separation between paralogs from *O. brachyantha*, *O. australiensis*, *O. officinalis*, *O. punctata*, *O. minuta *and Nipponbare, each paralogous set for these species forming a monophyletic group. However, neither the ML nor the MP trees allow clarification of the relationships between the copies of the older species, *P. parviflora, L. perrieri *and *O. granulata*. This analysis is complicated by the fact that we isolated only one paralog for *P. parviflora *and *O. granulata*. We observed a 221 bp repeat element insertion, accompanying a deletion in copy 2 of *O. brachyantha*, but no topology change was observed when excluding this large indel event before analysis. This repeat sequence belongs to the MITE castaway-like family (BLASTN against the TIGR-Oryza-repeat v3.3 database, e-value = 7.2e^-5^).

For gene H, we obtained peculiar results (Figure 4). The first obvious observation is the number and position of copies of the outgroup *L. perrieri*. We get at least 4 different copies, listed 1 to 4, respectively sister to *O. punctata 1*, *O. minuta 1*, *O. punctata 2*, and the clade regrouping *O. officinalis *and *O. minuta 3*. This result was checked by three independent cycles of cloning-sequencing, with two different *L. perrieri *DNA extracts. Only one copy was isolated for the most distant species *P. parviflora*, *O. brachyantha *and *O. granulata*, as well as for *O. officinalis*, and the two copies of *O. australiensis *are separated. However, both copies of *O. punctata *(if we except *L. perrieri *copies), *O. minuta*, and Nipponbare were closely related. As a result we have a mixed topology, with paralogous sequences evolving independently in the older species.

### Paralogous pair divergence

To investigate potential bias in paralog divergence, we first compared the sequence data sets (Table 2). The number of parsimony informative sites and indel events are given for information. The mean rates of synonymous (dS) and non-synonymous (dN) substitutions are the means for all sequence comparisons in each data set. Mean dS varies from 0.040 for sequence B to 0.150 for sequence F, mean dN varies from 0.010 for sequences C and H to 0.1 for sequence E, and mean K varies from 0.084 for sequence B to 0.236 for sequence F. There seems to be no correlation between the two kinds of observed topologies and the global divergence values of the data set, indicating that these genes are evolving at equivalent rates, whatever the proportion of within-species concerted evolution.

We show divergence values between each paralogous pair in Additional file 4. It would have been interesting to compute combined data set analysis, at least for a complete sampling matrix, in order to increase information support, but this was not possible as the paralogous pairs were not isolated for all species. We were particularly interested in the dS values, to examine global neutral evolution of our duplicated pairs, and the dN/dS ratio, to verify the neutrality hypothesis and detect signatures of positive selection. Mean dS values for paralogous pairs for each species ranged from 0.01 for *O. granulata *to 0.09 for *O. australiensis*, but there is a bias due to missing paralogs in some data sets. Paralogous dS rates were not significantly different (with p < 0.05, data not shown) between each species. Mean dS values for each gene ranged from 0.008 for gene G to 0.152 for gene E. dS rates were significatively higher for gene E, compared with genes B (Wilcoxon test, W = 0, p = 0.002), D (W = 0, p = 0.003) and G (W = 24, p = 0.013) at the 2.5% level. We observed the same difference between gene I and genes B (W = 6, p = 0.023), D (W = 4, p = 0.018) and G (W = 0, p = 0.014). These results are in agreement with the corresponding observed topologies. Mean dN/dS ratios for each paralogous pair ranged from 0.03 for *O. brachyantha *to 0.77 for *O. punctata*. Positive selection was tested between each pair in all genes with a Z-test of selection. Benjamini&Hochberg-corrected estimates of p-values were significant at the 0.05 probability level for three paralogous pairs: *O. sativa *ssp. *japonica *copies of gene B (dN-dS = 2.440, p = 0.0101), *L. perrieri *copies of genes B (dN-dS = 2.144, p = 0.02) and D (dN-dS = 2.049, p = 0.0261) and *P. parviflora *copies of gene G (dN-dS = 2.869, p = 0.00254).

The K ratio, the rate of nucleotide substitution calculated for orthologous non-coding sequences, is expected to be higher than the dN value and approximately equal to the dS rate, as non-coding sequences are also considered to evolve without selective pressure. However, if mechanisms leading to homogenization of paralogous pairs between both chromosomes 11 and 12 operate indiscriminately on both coding and non-coding sequences, we would expect that the intron sequences diverge more slowly between paralogs than between inter-species orthologs. If these mechanisms apply only to coding sequences, dN and dS rates between paralogs should be lower than K values, the latter showing no difference in paralogous and orthologous comparisons. Mean K values between paralogs for each gene vary from 0.034 for gene B to 0.247 for gene I, and seem to be correlated with the different topologies observed. We compared these data with divergence among the orthologs for each sequence. K substitutions were lower for paralogs than for orthologs for genes B (Wilcoxon test, W = 55, p = 0.003), D (W = 39, p = 0.027), H (W = 78, p = 0.001) and I (W = 210, p = 4.7e^-5^). The mean K value for all pairwise paralog comparisons was approximately 0.1 and was significantly lower than the mean K (0.1741) for all ortholog comparisons (Z-test, Z = 6.32, p = 7.034e^-9^). For comparison, K values calculated for adh orthologs (1766 bp in introns, data set extracted from Ge *et al*. [27]) varied from 0.035 (*O. australiensis-O. alterniflora*) to 0.338 (*O. brachyantha*- *L. perrieri*) with a mean of 0.185. We compared K, dS and dN mean ratios between paralogs, except for genes E and I, which present a topology of "duplication before speciation" type. Mean K was not significantly different from mean dS (Z-test, Z = 1.3, p = 0.067) and mean dN (Z-test, Z = 0.66, p = 0.106) at the 0.05 significance level. These data are more in favor of a homogenized concerted evolution mechanism along the whole genes and confirm results from Wang *et al*. [23], who described whole-gene conversion for two paralogous pairs of this duplication 11-12 in *O. sativa *ssp. *japonica *subspecies.

### Evolutionary dynamics of duplicated genes in *O. minuta*

In polyploid species, the evolution rates between duplicated copies are expected to change, either by accumulation of deleterious mutations in one of the redundant copies, leading to pseudogenization, or accumulation of positive mutations leading to neofunctionalization, or possibly subfunctionalization. Four copies for each sequence in the 11-12 duplication should be present in the tetraploid species *O. minuta*, two from the B genome and two from the C genome [27], except if gene loss has occurred early in the diploidization process. Thus, genes are three times redundant and we assessed whether this redundancy can influence their evolution. We tested to see (1) if we could detect accumulation of mutations and positive selection due to relaxed selection constraint or (2) if concerted evolution also homogenized all the homeologous copies. We isolated 3 copies for genes D, E and H and 4 for genes B and I. The divergence rates of the tetraploid copies were estimated by concatenating 5 sequences B, D, E, H, I for *O. punctata *1(BB), *O. punctata *2 (BB) and *O. officinalis *2 (CC) (taking the single copy of *O. officinalis *for sequence H), and *O. minuta *1 and 2 (subgenome BB) and 3 (subgenome CC). This yielded a total data matrix of 4167 bp, including 1043 bp in exons. We calculated the dN, dS and K ratios (Table 3) between each *O. minuta *copy and its orthologs in the diploid genomes, between the paralogous and paleologous copies themselves and, finally, between the surrogate diploid progenitors.

**Table 3:** Divergence rate of *Oryza minuta *copies

	dS	dN	dN/dS	K
*O. punctata 1/O. minuta 1*	0.000	0.007	/	0.0484
*O. punctata 2/O. minuta 2*	0.003	0.011	3.667	0.0525
*O. officinalis 2/O. minuta 3*	0.024	0.021	0.875	0.08
*O. minuta 1/O. minuta 2*	0.024	0.023	0.958	0.0815
*O. minuta 1/O. minuta 3*	0.045	0.043	0.956	0.0742
*O. minuta 2/O. minuta 3*	0.035	0.037	1.057	0.0873
*O. punctata 1/O. punctata 2*	0.021	0.033	1.571	0.0805
*O. punctata 1/O. officinalis 2*	0.035	0.036	1.029	0.1141
*O. punctata 2/O. officinalis 2*	0.035	0.035	1.000	0.079

	Ns	Nn		
		
*O. punctata 1*	8	25		
*O. punctata 2*	8.5	31.5		
*O. minuta 1*	8	26		
*O. minuta 2*	10	27		
*O. minuta 3*	11	36		
*O. officinalis 2*	8	29		

Synonymous (dS), non-synonymous (dN) and intronic (K) substitution rates are indicated between the allotetraploid *O. minuta *combined copies (from the five genes B, D, E, H and I), and their putative orthologs in diploid progenitors (*O. punctata *and *O. officinalis*), between the homeologous copies in *O. minuta*, and between the diploid parental orthologous copies. Number of synonymous (Ns) and non-synonymous substitutions (Nn) are indicated for the tetraploid and its parental genomes, with Nipponbare as outgroup.

Divergence (dN and dS) between *O. punctata *and *O. officinalis *copies on the one hand and *O. punctata *paralogs on the other are very similar, which could be explained by the close relationships between the two putative progenitors. dS values between these two species in the *MONOCULM1 *region were also low [25]. dS and dN ratios between *O. minuta *copies 1 and 2 (BB) were slightly lower than between copies 1 and 3 and copies 2 and 3. We postulate that if there was divergence of *O. minuta *copies from the parental copies, following by concerted evolution between the allotetraploid copies, the divergences observed now between *O. minuta1*/*O. minuta2 *and *O. minuta1*/*O. minuta3 *should be lower than between *O. punctata1*/*O. minuta1*, *O. punctata2*/*O. minuta2 *and *O. officinalis*/*O. minuta3*. Copies of the tetraploid and their respective diploid orthologs displayed very low substitution rates, in particular for *O. punctata *and *O. minuta*. This is more in favor of maintenance and parsimonious divergence of all the copies after the hybridization/polyploidization event than a concerted evolution of these copies. Concerning the dN/dS ratio, positive selection was only detected between *O. punctata *1 and *O. minuta 1 *copies (dN-dS = 2.307, p = 0.011). The *O. punctata 2-O. minuta 2 *pair presents a high dN/dS (3. 667), but the test was not significant (p = 0.054).

To compare with the data of Lu *et al*. [25], we calculated the number of synonymous and non-synonymous substitutions in the tetraploid and its parental genomes, with Nipponbare (copies 1 and 2) as outgroup (Table 3). Lu *et al*. showed that both non-synonymous and synonymous substitutions were in excess in *O. minuta*. Four of the 8 genes they tested had dN/dS >1 between *O. minuta *and the diploid progenitors, revealing relaxed pressure of selection in the tetraploid. The similar number of substitutions in the diploids and the tetraploids and detection of positive selection for only one of the allotetraploid copies in the duplicated 11-12 fragment are in favor of concerted evolutionary dynamics.

### Analysis of the duplicated region in *Sorghum *and *Brachypodium*

**Figure 5:**
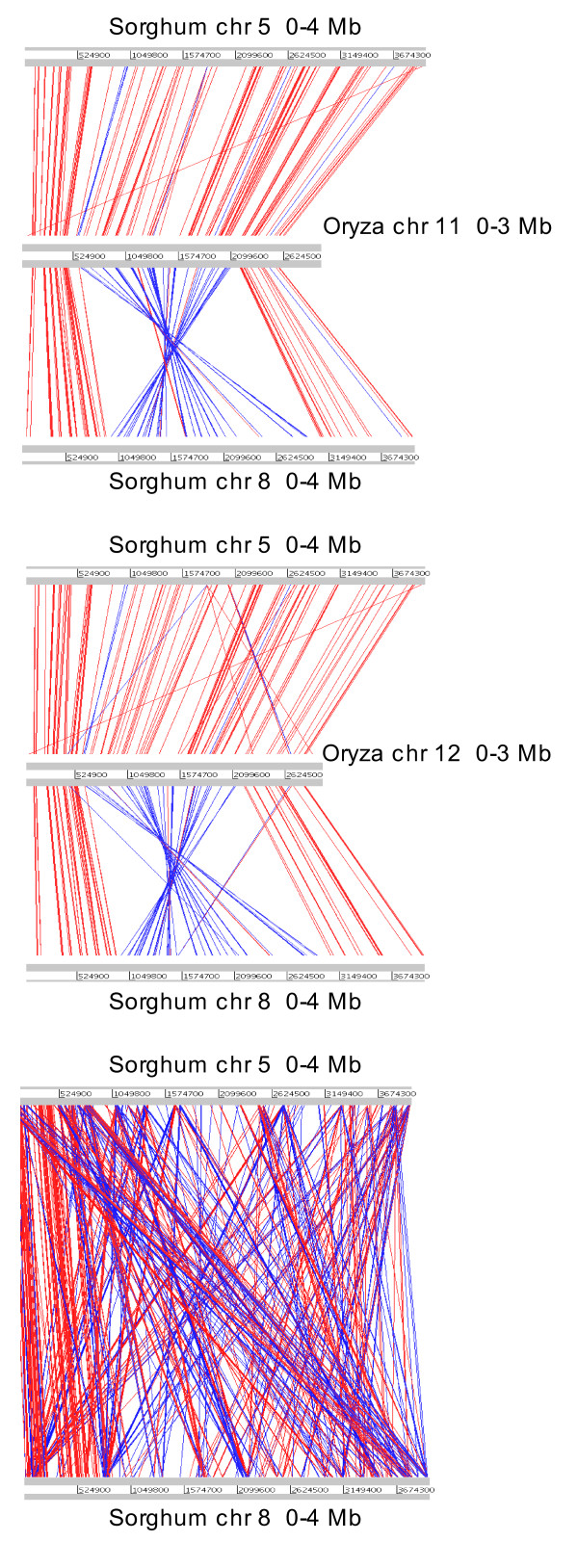
**Graphical representation of the syntenic regions between rice and sorghum**. Synteny relationships between the first 3 Mbp on rice chromosomes 11 and 12 and the first 4 Mbp on *Sorghum bicolor *chromosomes 5 and 8. Lines represent sequence similarity comparison by BLASTN. Each red line corresponds to a single match, with blue lines representing inverted matches. The minimum size and the minimum blast score of the matches displayed are 200 bases, except for comparison with sorghum chromosomes 5 and 8 (500 bases).

**Figure 6:**
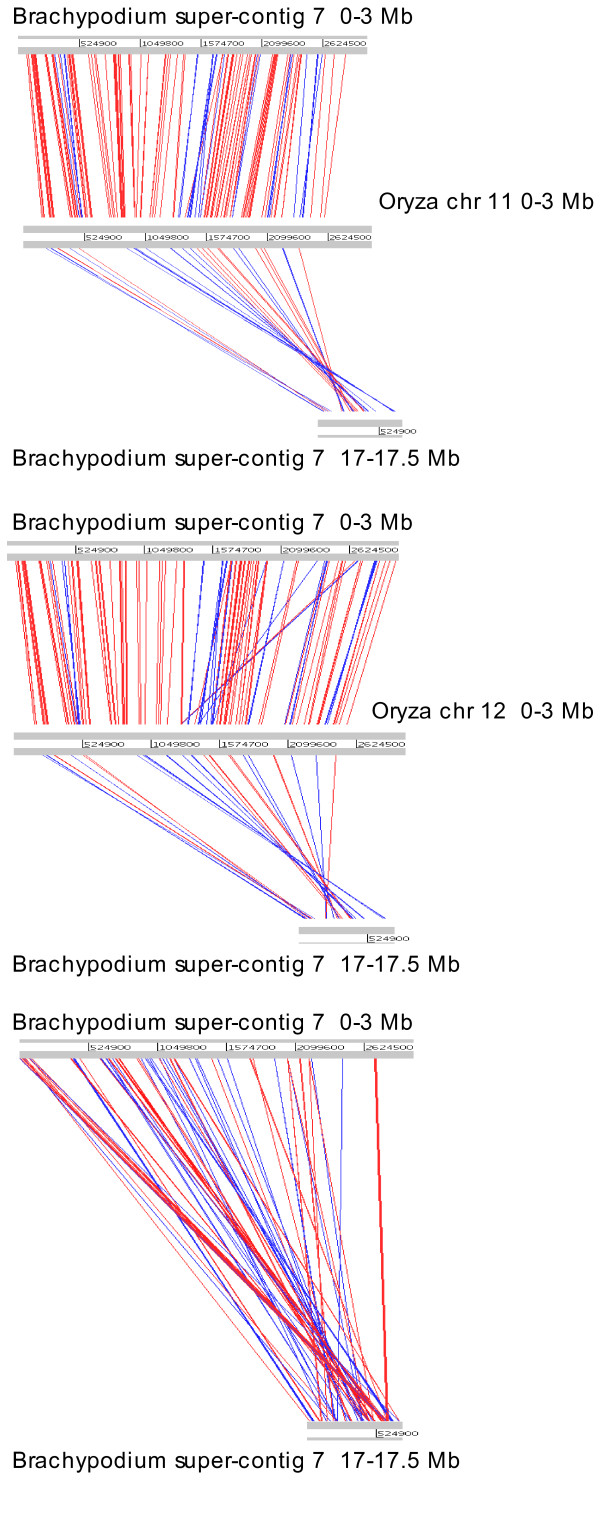
**Graphical representation of the syntenic regions between rice and *Brachypodium***. Synteny relationships between the first 3 Mbp on rice chromosomes 11 and 12 and the first 3 Mbp and last 0.5 Mbp on *Brachypodium distachyon *super-contig 7. Lines represent sequence similarity comparison by BLASTN. Each red line corresponds to a single match, with blue lines representing inverted matches. The minimum size and the minimum blast score of the matches displayed are 200 bases.

The amplification of two copies for most genes we selected in the 11-12 region, not only for species from the *Oryza *genus, but also from the related *Leersia perrieri *and *Potamophila parviflora*, was concordant with the recent results of Paterson *et al*. [26]. These authors detected a duplicated segment, also showing strong conservation, in the corresponding regions of *Sorghum bicolor *chromosomes 5 and 8 and suggested that the duplication event occurred before the cereal divergence. We used the Artemis comparison tool (ACT, see Methods section) to compare the 11-12 region with the sorghum chromosome sequences and look for evidence of conservation of the duplicated region in the new grass model species, *Brachypodium distachyon *http://www.brachypodium.org. BLAST analysis indeed showed strong similarity between the 3 Mbp region on rice chromosomes 11 and 12 and a 4 Mbp region on chromosomes 5 and 8 of sorghum. Surprisingly, there is a clear inversion of ~0.8 Mbp only on sorghum chromosome 8 between 1 and 1.8 Mbp which corresponds to 1.2 to 2 Mbp on rice chromosomes 11 and 12 (Figure 5). Sequence comparison with the current assembly of the 4× coverage of the *Brachypodium distachyon *genome identified only one contig, super-contig 7 (~17.7 Mbp). However, closer inspection showed that these hits corresponded to two different regions of this contig, the first 3 Mbp and the last 0.5 Mbp. ACT visualization of sequence conservation shows that the duplicated region at the end of the contig (beginning at 17 Mbp) is inverted compared with the sequences of chromosomes 11 and 12 (Figure 6).

## Discussion

The rice genus underwent two episodes of rapid diversification [28] and thus rapid speciation which, with the fact that the 11-12 subtelomeric region is highly conserved, explains the poorly resolved internal node in some of our trees. This leads to unclear phylogenetic relationships between *Oryza *species and the outgroup *Leersia perrieri*, in contrast to the observations of Guo and Ge [20]. Moreover, *Leersia *presents similar characteristics to *O. brachyantha*, which is on the boundary of the genus [29]. We identified more than two copies of the H sequence for *L. perrieri*, each sister to one *Oryza *species copy. *L. perrieri *was identified as a diploid species (2n = 24) [30], and we have obtained independent confirmation (A. d'Hont, personal communication). Two copies of Adh2 and Gpa1 were also isolated in this species [20], both of "*Leersia*" type. These genes and gene H may have been duplicated since the divergence of *Leersia *from the other *Oryza *branches but more sequence information from this species is necessary to draw precise conclusions. While we cannot exclude mechanisms of "birth and death" in the generation of new gene copies elsewhere in the wild species' genomes, our approach, including amplification on mapped BAC clones in all *Oryza *species, strongly suggests that the gene copies are effectively on the orthologous regions of these genomes.

Isolation of paralogous pairs in seven *Oryza *species and two outgroups confirmed, firstly, that the duplication is not specific to the genus and, secondly, that the gene sequences are highly conserved between species. Wang *et al*. [23] described a high level of concerted evolution in this duplication in the two *Oryza sativa *subspecies, *japonica *and *indica*, which they dated to 5-7MYA, but showed that this conservation was heterogeneous along the segment. Similarly, our analysis shows different phylogenetic topologies throughout the duplication in the *Oryza *genus. All species display the same evolutionary mechanisms for the first sequences on the duplication, with a "duplication after speciation" topology. While we cannot formally exclude independent duplication in all species, widespread concerted evolution is the most parsimonious explanation. Paralogous pair divergence is similar, showing high conservation of the sequences. Even the allotetraploid species, *Oryza minuta*, shows no evidence of relaxed selective pressure, despite the putative presence of four copies of each gene. This conservation throughout the genus and in related species suggests that concerted evolution in this subtelomeric region is a recurrent process. Moreover our analysis of the K ratio between paralogous and orthologous copies indicated that the concerted mechanism involved would occur on the whole genes, and not only on the coding sequences.

Recently, Paterson *et al*. [26] described a duplicated segment in the corresponding regions of the sorghum genome and suggested that the apparent segmental duplication in *Oryza sativa *resulted from the older pan-cereal duplication. These observations and our results indicate that we are no longer looking at the short-term evolution of recently-duplicated genes, as has been suggested [8,13,16,17], and that previous dating based on molecular clock calculations were biased by the weak divergence rate. However, these authors describe a much larger conserved, duplicated region in rice and the exact extent and degree of conservation remain to be determined. Our results rather suggest that recurrent gene conversion is probably limited to a relatively short region, with much higher conservation in the immediate sub-telomeric region and a gradient of sequence divergence. This may explain the relatively high divergence times (17MYA for rice/rice duplicates and 34MYA in sorghum) calculated by Paterson *et al*. [26]

In this context, a similar duplication in the *Brachypodium distachyon *genome is expected. Indeed, *Oryza *and *Brachypodium *both belong to the BEP (Bambusideae-Ehrhartoideae-Pooideae) clade, whereas sorghum belongs to the PACC (Panicoideae-Arundinoideae-Chloridoideae-Centothecoideae) clade [31]. These clades diverged between 50 and 70 MYA [19], soon after the divergence of the grasses. We identified two regions orthologous to the 11-12 duplication on the first *Brachypodium *genome release, confirming its presence in this species, although future assemblies using deeper coverage will be needed to confirm the chromosome locations.

Gene conversion and unequal crossing-over events are the mechanisms proposed to explain such a level of conservation after tens of millions of years, but more in-depth genomic and cytological work would help to determine the type and frequency of these events. An inversion event, which constitutes a major chromosomal locus rearrangement, was detected on sorghum chromosome 8 and potentially in one of the *Brachypodium *(end of the super-contig 7) duplicated regions. Inversions can be a source of genomic novelties as well as sequence divergence [32] and such an event in a region which has undergone concerted evolution suggests that it is probably recent.

In the more proximal region of the duplication (genes F, H and I), gene pairs appear to be less influenced by concerted evolution as we observed "duplication before speciation" topologies and isolated single copies for ancient species. Moreover the neutral dS rate was stronger for these genes. This could be explained either by divergence of one of the sequences, making amplification of both copies with primers designed on *Oryza sativa *impossible, or loss of one copy, as for the majority of duplicated genes in rice through the diploidization process [8]. A clear rupture in highly-conserved colinearity can be observed in the dot plot of the 11-12 region in *Oryza sativa *(Additional file 1). Wang *et al*. [23] proposed a first model of the distribution and order of crossing over events throughout the duplication explaining the heterogeneity in sequence similarity between *japonica *paralogs. We will be able to extend this model to wild species with finer genome analysis, but our results on gene H (L-Galactono, 4-lactone dehydrogenase) already suggest recent conversion events specific to two species (*O. sativa *and *O. punctata*).

Genetic recombination is influenced by chromosomal location [33]. The subtelomeric location of the 11-12 duplication could be one factor explaining its evolution. However, the subtelomeres of rice have rather been described as dynamic regions where duplications have spawned new copies of genes [34]. In agreement with our observations, Wang *et al*. [35] recently described gene conversion occurring at a higher frequency towards the terminal regions of rice and sorghum chromosomes, showing wholly converted genes at an average distance of 3 Mbp from the telomeres in rice and a similar tendency in homologous regions of sorghum. However, these calculations are biased by the over-representation of two duplicated regions, between chromosomes 3 and 10 and the 11-12 duplication, which represent between them 82% of wholly converted genes and, to a lesser extent, high levels of conversion in the orthologous regions in sorghum. Rather than being a genome-wide phenomenon, these observations suggest that as-yet unknown selective pressures have contributed to the maintenance of high sequence identity within these two specific regions, and particularly the 11-12 duplication.

Our results suggest the presence of two duplicated chromosomal fragments, currently found on all *Oryza *chromosomes 11 & 12, sorghum chromosomes 5 & 8, and *Brachypodium *contig 7, which have been homogenized through concerted evolution since the ancestral WGD, dated after the Eudicot-Monocot divergence (between 150 and 200 MYA [36,37]). Wang *et al*. [23] proposed a stochastic evolution of gene pairs, in which conversion acts as an occasional, sometimes frequent interruption to independent evolution of paralogs. Our observations on genes in the subtelomeric 11-12 region throughout the *Oryza *genus and in related species, suggesting continuous concerted evolution affecting the same gene pairs in widely-divergent species, are not in agreement with this hypothesis. As suggested above, they rather indicate mechanisms acting preferentially in specific duplicated regions, and most notably in the duplication between chromosomes 11 and 12.

## Conclusions

Our observations suggest recurrent but heterogeneous concerted evolution has led to the extremely high sequence conservation occurring in this region of more than 2 Mbp. The detection of paralogous copies for almost all genes in all the species studied indicates a specific mechanism which has led to conservation in this duplicated region throughout the *Oryza *genus and in related species. It will be interesting to compare detailed structure of both distal ends of chromosomes 11 and 12 with other rice genomic regions (chromosomes 3 and 10). More detailed comparative analysis will allow a clearer understanding of the selection or structural pressure which tends to conserve this particular region.

## Methods

### Species sampling and amplification

Among the 23 species of the genus *Oryza*, representing 6 diploid genome types and 4 allotetraploids, we included 6 diploid species; *O. sativa japonica *(AA), *O. punctata *(BB), *O. officinalis *(CC), *O. australiensis *(EE), *O. brachyantha *(FF), *O. granulata *(GG) and a tetraploid *O. minuta *(BBCC). We also included two closely-related species, *Leersia perrieri *and *Potamophila parviflora*. Information on the samples used for phylogenetic reconstruction is displayed in Figure 1.

Translations of sequences annotated as coding sequences from genes in the first 2.5 Mbp of chromosomes 11 and 12 were used to isolate informative paralogous genes on the Nipponbare genome from the Rice Annotation Genome database [38]. These sequences were aligned with all *O. sativa japonica *cDNA sequences using TBLASTN [39] at an e-value of 10^-5 ^to select only genes for which there is proof of expression. The corresponding coding sequences were used to perform a BLASTN search against the combined Oryza Map Alignment Project (OMAP [40]) BAC-end libraries. These libraries, representing 11 genomes of wild species in the *Oryza *genus, provide comprehensive coverage (at least 5×) of these genomes. Alignments with the most distant *Oryza *species were used as targets for primer design, choosing primers which were specific to the cognate genes on chromosomes 11 and 12 in the *O. sativa *genome and amplified no other target. We designed 22 pairs of primers for amplifying orthologous segments from all *Oryza *species, among which nine genes were selected on the basis of copy number (only two copies for most pairs in diploid genomes; exceptions are noted in the Results section), their distribution along the conserved region and their length (minimum of 200 bp). The presence of the genes on the orthologous chromosomes of the wild rice species was confirmed by amplification on DNA from BAC clones which have been mapped by FingerPrinted Contigs and sequence comparison of BAC-ends to the orthologous chromosomes in the *Oryza *species. Information on the nine paralogous pairs is given in Table 1.

Sequences were PCR amplified in a 25 μl reaction comprising 5 μl GoTaq Tp5x buffer, 2.5 μl dNTPs (2.5 mM solution), 0.20 μl GoTaq polymerase (5 u/μl), 0.65 μl of each primer (10 μM) (See Additional file 5: Primers and hybridization temperatures), 1 μl DNA, and 15 μl H_2_O. PCR cycling consisted of 30 cycles of 1 min at 94°C, 45 sec annealing at each sequence annealing temperature (Additional file 5), and 1 min 30 sec at 72°C. All amplified fragments were cloned into the pGEM T-easy vector (Promega, Madison, WI, USA). Plasmid DNA was extracted with a ProMega (Madison, WI, USA) kit and sequenced on both strands on an Applied Biosystems (Foster City, CA, USA) ABI Prism 3130XL sequencer using universal primers. To isolate all gene copies we cloned approximately 10 to 20 clones for diploid species and 15 to 40 for the tetraploid *O. minuta*. Genbank accession numbers for each sequences are listed in Table 2.

### Phylogenetic analysis

Sequences were first aligned with Muscle [41] then refined manually in the data matrix using Seaview [42] and Bioedit http://www.mbio.ncsu.edu/BioEdit/BioEdit.html. Maximum parsimony analyses were performed on PAUP [43], using the Heuristic algorithm with default parameters. Analyses were conducted either with insertions/deletions included or with indels excluded and recoded according to Simmons and Ochoterena [44]. We used PhyML 3.0 [45] for maximum likelihood analysis and the automated tool provided by the Datamonkey webserver [46] for selection of the DNA substitution model. For all the sequence data sets, the Hasegawa-Kishono-Yano (HKY85) model, which does not assume equal base frequencies and accounts for the difference between transitions and transversions with one parameter, was selected. Bootstrap support was estimated with 1000 bootstrap for both methods.

To characterize the matrix data set and the divergence of the paralogous copies, we calculated pairwise non-synonymous (dN) and synonymous (dS) nucleotide substitutions per sites in the coding regions with the modified Nei-Gojobori method [47] in MEGA4, with overall transition/transversion bias for each CDS also estimated in MEGA4 [48]. We define the number of substitutions per site in the non-coding regions (introns) as the K rate. It was calculated with DnaSP [49], along with the deletion/insertion polymorphism (DIPs). dS and K variation was tested with a Wilcoxon test, with α = 0.05, and mean K was tested with a parametric Z-test. Selective pressure (dN/dS >1) was tested using a Codon-Based Z-test in MEGA4, with all positions containing alignment gaps eliminated in pairwise sequence comparisons. Significance levels were corrected for multiple tests (672 comparisons overall) for diploid analyses using the Benjamini&Hochberg procedure [50].

### Sequence analyses

Dot plots were carried out with the Dotter programme [51] using default parameters. Syntenic regions were identified by TBLASTN alignment against *Sorghum bicolor *[26] and *Brachypodium distachyon *(first public release, http://www.brachypodium.org/) genomic sequences with a cut-off of e^-15 ^using all CDS on the duplicated region (3 Mbp) on chromosomes 11 (546 CDS) and 12 (548 CDS). Large-scale sequence conservation was analysed using the Artemis Comparison Tool (ACT [52]) to project BLASTN alignments carried out on the Health Protection Agent Double Act server http://www.hpa-bioinfotools.org.uk/pise/double_act.html, after masking repeated sequences with RepeatMasker [53]. Dot-plots on subsequences of 1 Mbp were used to detect small-scale rearrangements.

## Abbreviations

CDS: coding sequence; dN: non-synonymous substitution rate; dS: synonymous substitution rate; Mbp: megabase pairs; MITE: miniature inverted-repeat transposable element; ML: maximum likelihood; MP: maximum parsimony; MYA: million years ago; WGD: whole genome duplication

## Authors' contributions

JJ participated in the design of the study, carried out the molecular biology studies, performed the phylogenetic, comparative genomic and statistical analysis, and drafted the manuscript. ML participated in the design of the study and acquisition of sequence data. RC conceived the analysis, participated in its design, and helped to draft the manuscript. All authors read and approved the final manuscript.

## Supplementary Material

Additional file 1 **Dot plot of the subtelomeric regions of rice chromosomes 11 (horizontal) and 12 (vertical)**. Analysis was as described in Methods. The zoom represents the region overlapping the rupture of colinearity at ~1.8 Mbp up to ~2.5 Mbp. Click here for file

Additional file 2 **ML trees inferred from genes A (1) and C (2)**. These genes were amplified on *Oryza brachyantha*, *O. granulata*, *Leersia perrieri *and *Potamophila parviflora*. The same topology "duplication after speciation" was obtained. Numbers above branches indicated bootstrap support of ML and MP respectively. If only one number is present, that means incongruence between the two methods and only the ML bootstrap is shown Click here for file

Additional file 3 **ML trees inferred from genes F (3) and G (4)**. These genes were amplified on *Oryza brachyantha*, *O. granulata*, *Leersia perrieri *and *Potamophila parviflora*. The same topology "duplication after speciation" was obtained. Numbers above branches indicated bootstrap support of ML and MP respectively. If only one number is present, that means incongruence between the two methods and only the ML bootstrap is shown Click here for file

Additional file 4 **Divergence between paralogous pairs**. Numbers of synonymous substitutions (syn), non-synonymous substitutions (nonsyn), synonymous (dS) and non-synonymous (dN) substitutions rates, substitutions in intronic regions (subst/intron), intronic substitutions rate (K) and total polymorphism (Polymorphism), which sums the syn + non/syn + subst/intron, are displayed for each species paralogous pairs and for each sequences. On the right and at the bottom are mean values. Click here for file

Additional file 5 **Primers and hybridization temperatures**. The genes in bold characters were amplified on the complete sample set whereas the others were amplified on the more distant species only. Click here for file
