# Supplementary figures and images for: A recent duplication revisited: phylogenetic analysis reveals an ancestral duplication highly-conserved throughout the Oryza genus and beyond
Source: BMC Plant Biol. 2009 Dec 10;9:146. doi: 10.1186/1471-2229-9-146 (PMC2797015; doi:10.1186/1471-2229-9-146)

(1)

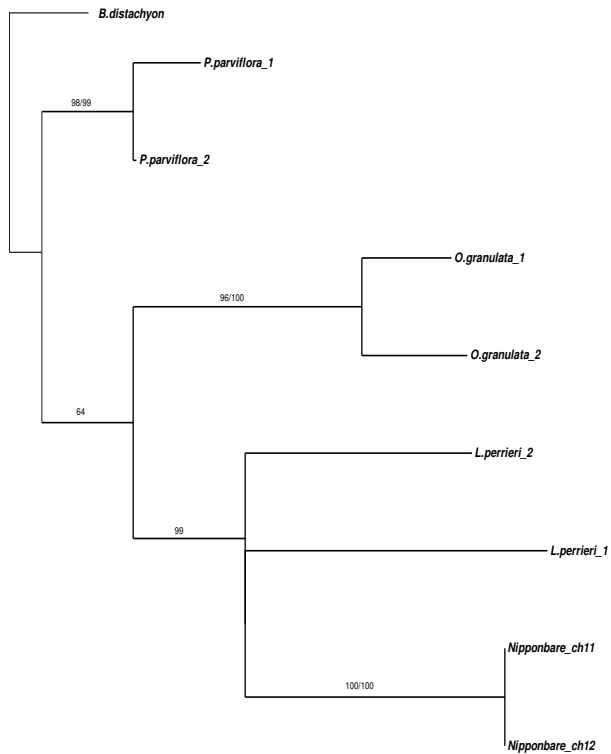

(2)

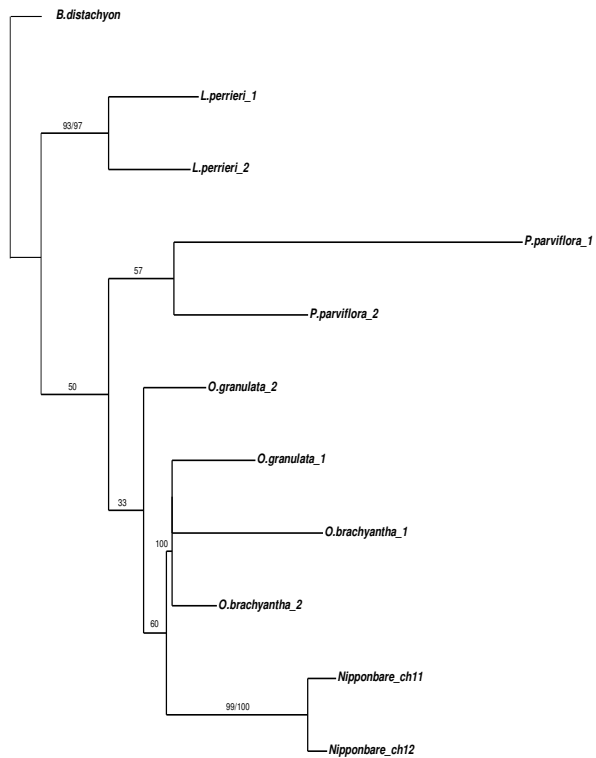

Supplement: Additional file 2 — ML trees inferred from genes A (1) and C (2). These genes were amplified on Oryza brachyantha, O. granulata, Leersia perrieri and Potamophila parviflora. The same topology "duplication after speciation" was obtained. Numbers above branches indicated bootstrap support of ML and MP respectively. If only one number is present, that means incongruence between the two methods and only the ML bootstrap is shown [file 1471-2229-9-146-S2.PDF]

(3)

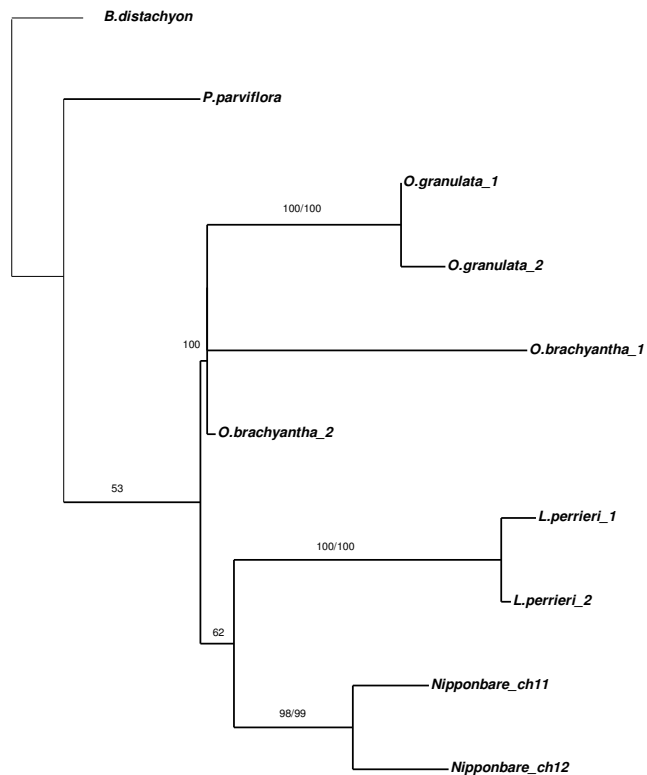

(4)

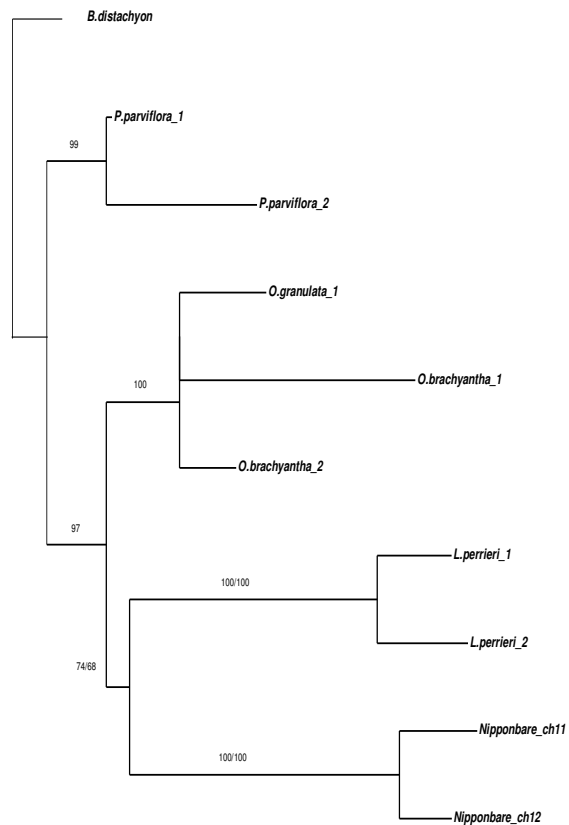

Supplement: Additional file 3 — ML trees inferred from genes F (3) and G (4). These genes were amplified on Oryza brachyantha, O. granulata, Leersia perrieri and Potamophila parviflora. The same topology "duplication after speciation" was obtained. Numbers above branches indicated bootstrap support of ML and MP respectively. If only one number is present, that means incongruence between the two methods and only the ML bootstrap is shown [file 1471-2229-9-146-S3.PDF]
