# Supplementary material for: A recent duplication revisited: phylogenetic analysis reveals an ancestral duplication highly-conserved throughout the Oryza genus and beyond
Source: BMC Plant Biol. 2009 Dec 10;9:146. doi: 10.1186/1471-2229-9-146 (PMC2797015; doi:10.1186/1471-2229-9-146)
Supplement: Additional file 4 — Divergence between paralogous pairs. Numbers of synonymous substitutions (syn), non-synonymous substitutions (nonsyn), synonymous (dS) and non-synonymous (dN) substitutions rates, substitutions in intronic regions (subst/intron), intronic substitutions rate (K) and total polymorphism (Polymorphism), which sums the syn + non/syn + subst/intron, are displayed for each species paralogous pairs and for each sequences. On the right and at the bottom are mean values. [file 1471-2229-9-146-S4.PDF]

		Nipponbare	*O.punctata*	*O. officinalis*	*O. australiensis*	*O. brachyantha*	*O. granulata*	*L. perrieri*	*P. parviflora*	***Mean***
A	syn	0					0	6	1	**1.750**
	Nsyn	0					1	1	0	**0.500**
	dS	0.000					0.000	0.137	0.023	**0.040**
	dN	0.000					0.011	0.011	0.000	**0.006**
	dN/dS	/					/	0.080	0.000	**0.040**
	subst/intron	0					6	11	2	**4.750**
	K	0.000					0.099	0.192	0.031	**0.080**
	Polymorphism	0					7	18	3	**7.000**
B	syn	0	1	3	1	0	3	0	3	**1.375**
	nonsyn	4	3	3	3	0	10	4	2	**3.625**
	dS	0.000	0.020	0.060	0.025	0.000	0.061	0.000	0.060	**0.028**
	dN	0.032	0.024	0.024	0.024	0.000	0.078	0.031	0.016	**0.029**
	dN/dS	/	1.200	0.400	0.980	/	1.279	/	0.267	**0.825**
	subst/intron	5	9	14	8	3	13	15	14	**10.125**
	K	0.016	0.030	0.047	0.026	0.010	0.043	0.050	0.047	**0.034**
	Polymorphism	9	13	20	12	3	26	19	19	**15.125**
C	syn	0				3	0	1	4	**1.600**
	nonsyn	0				0	0	0	1	**0.200**
	dS	0.000				0.113	0.000	0.038	0.149	**0.060**
	dN	0.000				0.000	0.000	0.000	0.016	**0.003**
	dN/dS	/				0.000	/	0.000	0.107	**0.036**
	subst/intron	4				2	7	6	17	**7.200**
	K	0.059				0.029	0.106	0.090	0.288	**0.114**
	Polymorphism	4	0	0	0	5	7	7	22	**5.625**
D	syn	5	2	2		1	1	0	5	**2.286**
	nonsyn	1	3	4		0	0	4	5	**2.429**
	dS	0.058	0.023	0.023		0.012	0.012	0.000	0.058	**0.027**
	dN	0.005	0.015	0.020		0.000	0.000	0.019	0.024	**0.012**
	dN/dS	0.086	0.652	0.870		0.000	0.000	/	0.414	**0.337**
	subst/intron	1	7	3		0	0	7	5	**3.286**
	K	0.009	0.144	0.029		0.000	0.000	0.069	0.048	**0.043**
	Polymorphism	7	12	9		1	1	11	15	**8.000**
E	syn	2	1	2	2			4	3	**2.333**
	nonsyn	1	2	3	1			4	4	**2.500**
	dS	0.100	0.100	0.200	0.201			0.201	0.110	**0.152**
	dN	0.061	0.073	0.128	0.121			0.131	0.136	**0.108**
	dN/dS	0.610	0.730	0.640	0.602			0.652	1.236	**0.745**
	subst/intron	48	39	75	77			90	59	**64.667**
	K	0.123	0.098	0.202	0.209			0.250	0.154	**0.173**
	Polymorphism	51	42	80	80	0	0	98	66	**52.125**
F	syn	6				1	0	0		**1.750**
	nonsyn	0				0	0	1		**0.250**
	dS	0.107				0.018	0.000	0.000		**0.031**
	dN	0.000				0.000	0.000	0.008		**0.002**
	dN/dS	0.000				0.000	/	/		**0.000**
	subst/intron	21				6	6	3		**9.000**
	K	0.139				0.037	0.037	0.018		**0.058**
	Polymorphism	27				7	6	4		**11.000**
G	syn	1				0		3	1	**1.250**
	nonsyn	2				2		2	7	**3.250**
	dS	0.016				0.000		0.016	0.000	**0.008**
	dN	0.005				0.011		0.011	0.032	**0.015**
	dN/dS	0.313				/		0.688	/	**0.500**
	subst/intron	8				6		6	3	**5.750**
	K	0.049				0.037		0.037	0.018	**0.035**
	Polymorphism	11	0	0	0	8		11	11	**5.857**
H	syn	4	9		6					**6.333**
	nonsyn	1	5		4					**3.333**
	dS	0.047	0.106		0.059					**0.071**
	dN	0.005	0.026		0.021					**0.017**
	dN/dS	0.106	0.245		0.356					**0.236**
	subst/intron	20	29		86					**45.000**
	K	0.038	0.056		0.181					**0.092**
	Polymorphism	25	43		96					**54.667**
I	syn	3	1	1	2	3		3		**2.167**
	nonsyn	4	3	0	3	1		2		**2.167**
	dS	0.118	0.040	0.039	0.079	0.120		0.117		**0.086**
	dN	0.054	0.041	0.000	0.041	0.014		0.027		**0.030**
	dN/dS	0.458	1.025	0.000	0.519	0.117		0.231		**0.392**
	subst/intron	75	58	50	61	107		114		**77.500**
	K	0.232	0.173	0.146	0.183	0.358		0.389		**0.247**
	Polymorphism	82	62	51	66	111		119		**81.833**
**Mean dS**		**0.05**	**0.06**	**0.08**	**0.09**	**0.04**	**0.01**	**0.06**	**0.07**	
**Mean dN**		**0.02**	**0.04**	**0.04**	**0.05**	**0**	**0.02**	**0.03**	**0.04**	
**Mean dN/dS**		**0.19**	**0.77**	**0.4**	**0.61**	**0.03**	**0.64**	**0.33**	**0.4**	
Mean K		**0.07**	**0.10**	**0.11**	**0.15**	**0.08**	**0.06**	**0.14**	**0.10**	
