# Supplementary material for: A recent duplication revisited: phylogenetic analysis reveals an ancestral duplication highly-conserved throughout the Oryza genus and beyond
Source: BMC Plant Biol. 2009 Dec 10;9:146. doi: 10.1186/1471-2229-9-146 (PMC2797015; doi:10.1186/1471-2229-9-146)
Supplement: Additional file 5 — Primers and hybridization temperatures. The genes in bold characters were amplified on the complete sample set whereas the others were amplified on the more distant species only. [file 1471-2229-9-146-S5.PDF]

| | Chr11 | Chr12 | Forward | Reverse | Ta |
|---|---|---|---|---|---|
| *A* | Os11g01154 | Os12g01160 | TTTGCCAGAACCTGCATTAGG | GATGAAGTAGAAACAGTAACAGG | 55 |
| *B* | Os11g01380 | Os12g01390 | GATTGAATCACAGAATGAAGTAAGG | CACACAACCTATACAAATAATGG | 55 |
| *C* | Os11g01420 | Os12g01430 | GACAAGGTACAGTCGGAATGTTGC | TATGTGAAGAAGGGAAGCCCATTTC | 60 |
| *D* | Os11g03050 | Os12g02820 | CGTGCTGTAAAATGGGTGCACGA | CATCAATTTTCTGGCCATGCCCG | 63 |
| *E* | Os11g03730 | Os12g03470 | GGAATCAGCCATAATGATGAAG | CTCTAACAGAACCCCATGTT | 54 |
| *F* | Os11g04030 | Os12g03860 | GGACAGAGGAAGATATTGGATTCTATG | CAGCCATCCAGTACTTCACACTTAATC | 57 |
| *G* | Os11g04200 | Os12g04010 | CCAAGAAAGTTCAATTCAAGGATGAAC | CAAGAGAAGCAGTGACTTCCTCAGTG | 58 |
| *H* | Os11g04740 | Os12g04520 | ACCAAGTCCACAGCGAGCAAG | GACGCCAAGAAGAAGACCGTCAC | 63 |
| *I* | Os11g04980 | Os12g04990 | GCCAGATCATCCATCAGCTTC | CCTTTAGCTCACATCTATGAGAGAG | 58 |
